# A novel strategy for conversion from pediatric V-A ECMO to CPB circuit

**DOI:** 10.1051/ject/2025027

**Published:** 2025-09-15

**Authors:** Amita Lal, David Machin, William Lansdowne

**Affiliations:** 1 Perfusion Department, Bristol Royal Hospital for Children Bristol United Kingdom

**Keywords:** ECMO, CPB, Pediatric, Cardiac surgery, Conversion

## Abstract

This article describes two novel strategies to convert a veno-arterial extracorporeal membrane oxygenator (V-A ECMO) supported pediatric patient circuit to a cardiopulmonary bypass (CPB) circuit. Modification of the existing ECMO circuit incorporated a venous reservoir, cardioplegia circuit, ultrafiltration circuit, and cardiotomy suckers, allowing all aspects of cardiac surgery to be performed. This approach eliminated the need for conversion to an additional CPB circuit, thereby reducing surface area exposure and blood product requirement. We found that these patients had no major post-operative coagulopathies or observable neurological dysfunction.

## Overview

The prevalence of pediatric heart disease in the UK is estimated at 1 in 133 babies every year, and globally, approximately 1 in 100 [1]. Of which, either surgical or interventional care is required in approximately 33–50% of cases [2]. The variety of pediatric congenital heart defects can be complex, and this can make the surgical intervention more complicated. Thus, post-operatively, some patients may require further support.

The purpose of V-A ECMO is to bridge the gap for these patients to support the heart and lungs in recovery or transplant. While on this modality, clinicians can assess the cardiac function and effectively treat these patients. However, it can be decided that further surgical cardiac intervention is needed. This requires the patient to be removed from the ECMO circuit and supported by the CPB circuit. However, this exposes the patient to an additional extracorporeal circuit, increasing native blood to foreign surface area exposure and its consequences [3]. Furthermore, the high CPB circuit volume to pediatric patient blood volume ratio requires additional blood products to prime the CPB circuit, which again has associated risks [4].

With the aforementioned concerns, we describe a modification of our existing ECMO circuit. Previous reports have shown similar modifications. However, in this report, the equipment used and the technique carried out were changed [5]. The amendments to our ECMO circuit provided the features of a CPB circuit by incorporating a venous reservoir, cardioplegia circuit, hemofiltration circuit, cardiotomy suckers, and safety devices. The technique was performed on two pediatric patients, supported on CentriMag^®^ V2 Console ECMO system (Abbott Cardiovascular, Maidenhead, UK), using either the Quantum CPB system (Spectrum Medical, Cheltenham, UK) or the Stockert s5 heart-lung machine (Sorin Group, Mirandola, Italy).

## Description

Two patients on V-A ECMO support required further cardiac surgery after ECMO was established. Both patients used the techniques described herewith.

Patient one was 5 days old, 3.3 kg male, with hypoplastic left heart syndrome, who required V-A ECMO post-Norwood procedure and returned to theatre 5 days post-operative for repair of the systemic aortic valve. This patient was converted from ECMO to CPB using the Quantum Modified ECMO System (MES).

Patient two was 8 days old, a 3.4 kg female, with Transposition of the Great Arteries, who required V-A ECMO support following an arterial switch operation. The patient returned to theatre for an aortic valve repair and main pulmonary artery enlargement and was converted from ECMO to CPB using the s5 MES.

### V-A ECMO to CPB conversion using Quantum MES

To convert the patient from ECMO to CPB, the Quantum MES and the CentriMag ECMO circuit were used simultaneously. In this configuration, the main pump flow was regulated by the CentriMag console and centrifugal pump of the ECMO circuit. The Quantum CPB system managed the ventilation control, safety features (bubble detector and arterial occluder), and frame for the different components. The initial setup of the Quantum CPB system used a primed Pixie reservoir (Medtronic, Watford, UK) and CSC 14 cardioplegia device (Sorin Group, Mirandola, Italy), given at a 4:1 cold blood configuration. The water lines were removed from the HILITE 2400 LT ECMO oxygenator (MEDOS, Medizintechnik AG, Stolberg, Germany), and the gas line was redirected to the Quantum Ventilation Module on the Quantum CPB frame. The ECMO oxygenator and CentriMag pump head were then moved simultaneously to the Quantum CPB frame, with the oxygenator remaining higher than the pump head. Water lines and the cardioplegia system were attached to the oxygenator, and the arterial line was placed in the tubing occluder. The circuit pressures and CDI^®^ 550 (Terumo, Leuven, Belgium) monitoring remained on the ECMO cart.

**Figure 1:**
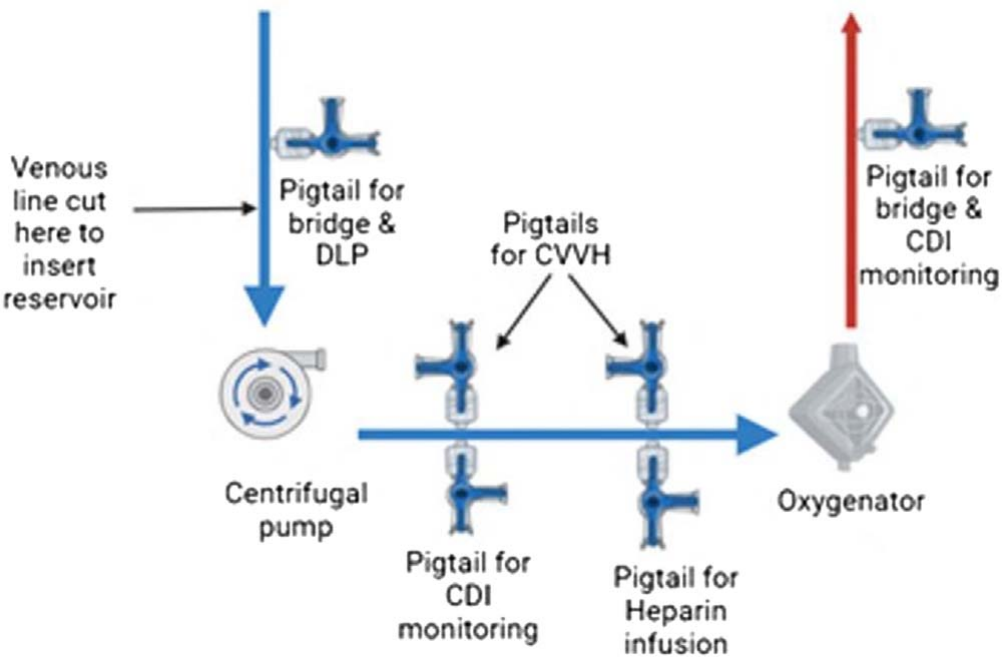
Bristol University Hospital for Children ECMO circuit design. CVVH: Continuous Veno-Venous Hemofiltration, DLP: Drainage line pressure.

**Figure 2:**
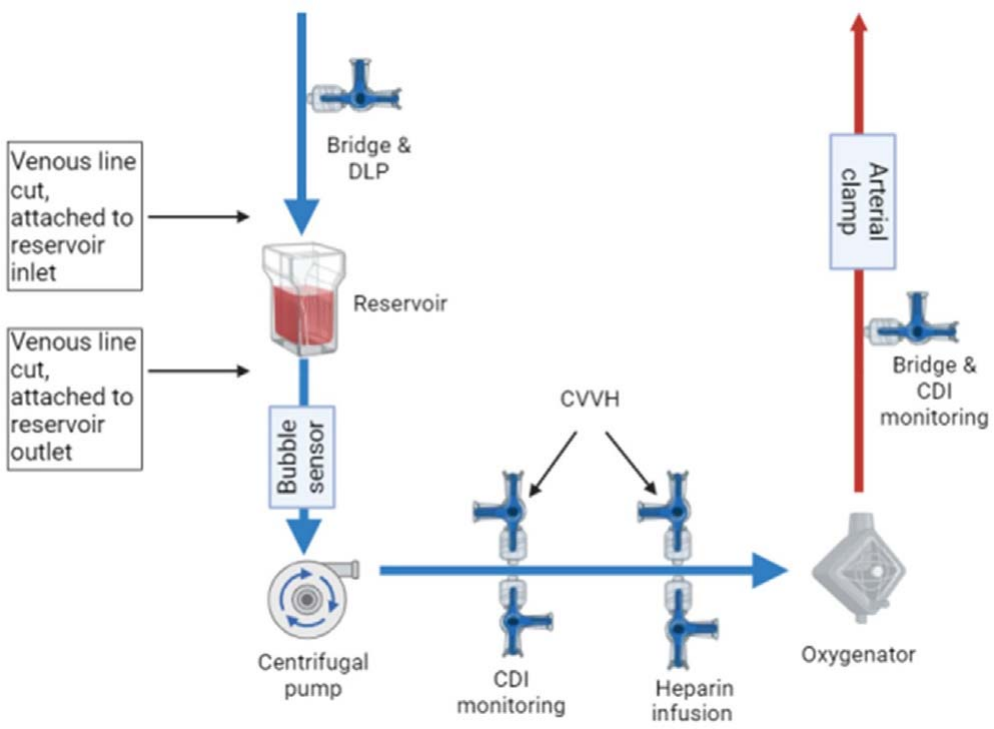
Quantum MES circuit design. DLP: Drainage line pressure, CVVH: Continuous Veno-Venous Hemofiltration.

**Figure 3:**
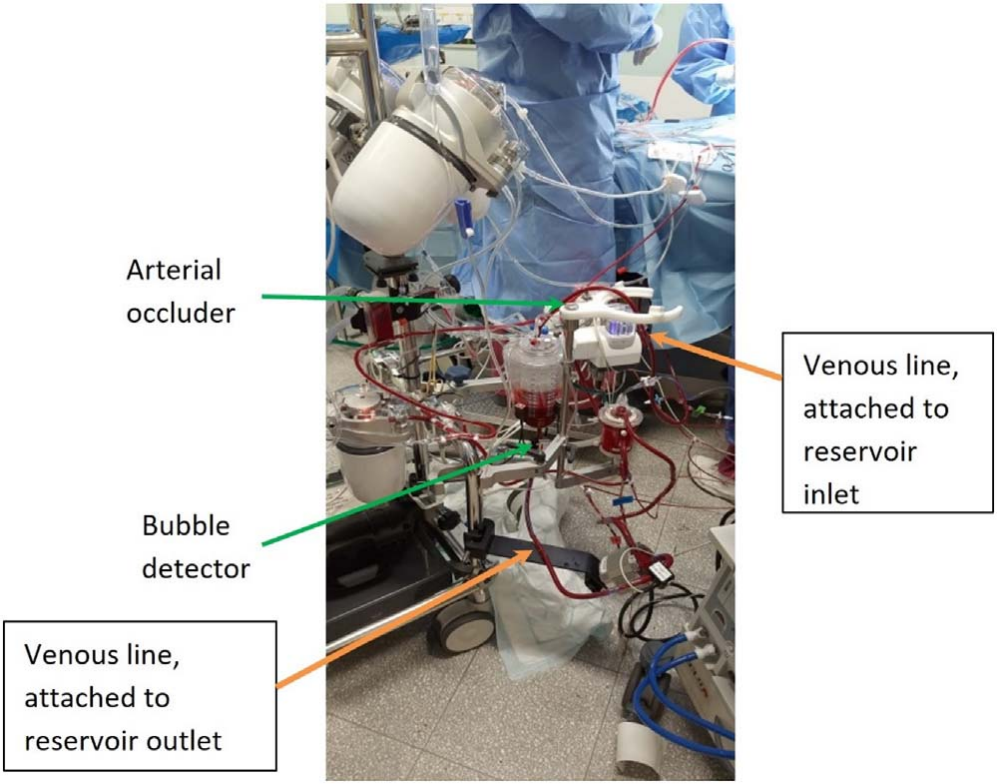
Quantum MES circuit technique.

In preparation for conversion to CPB, the patient was fully heparinized with a loading dose of 300 I.U./kg. The patient had standard cannulation, ascending aorta with an 8fr DLP™ One-Piece Pediatric Arterial Cannula (Medtronic, Watford, UK) and right atrium with 16fr DLP™ Single Stage Venous Cannulae with right angle (Medtronic, Watford, UK), the cannula remained for CPB. The patient was then converted to CPB in the following sequence: the pump head speed was reduced, the arterial line clamped, and the venous line double clamped in a predefined area (Fig. 1). Using aseptic technique, the venous line between the two clamps was cut and a Pixie venous reservoir inserted. The upper limb was connected to the reservoir inlet, and the lower limb to the reservoir outlet, ensuring an air-free connection. After both connections were made, the venous clamps were removed, the pump flow increased, and the arterial line was unclamped to initiate CPB (Figs. 2 and 3).

### V-A ECMO to CPB conversion using s5 MES

To convert the patient from ECMO to CPB, the s5 heart-lung machine (HLM) with the CentriMag ECMO circuit was used simultaneously, with the ECMO New Born A.L. One (Eurosets, Medolla, Italy) oxygenator. However, pump flow was controlled via a roller pump on the s5 HLM. In preparation for conversion to CPB, a primed Pixie reservoir, roller pump tubing, and CSC 14 cardioplegia device (given at a 4:1 cold blood configuration) were set up on the s5 HLM. This also regulated gas flow and safety features (bubble and level sensor). All other components remained on the ECMO cart.

**Figure 4:**
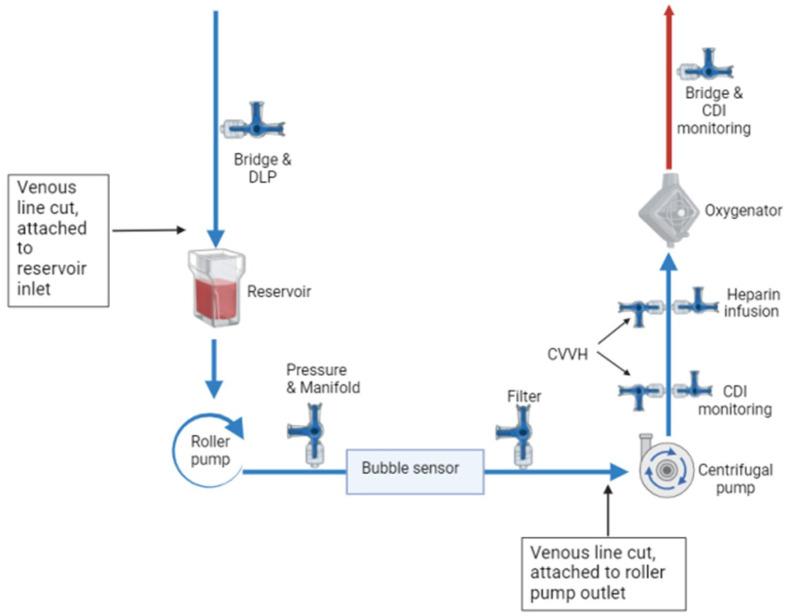
s5 MES circuit design. DLP: Drainage line pressure, Filter: CPB hemofilter for conventional and modified ultrafiltration, CVVH: Continuous Veno-Venous Hemofiltration.

**Figure 5:**
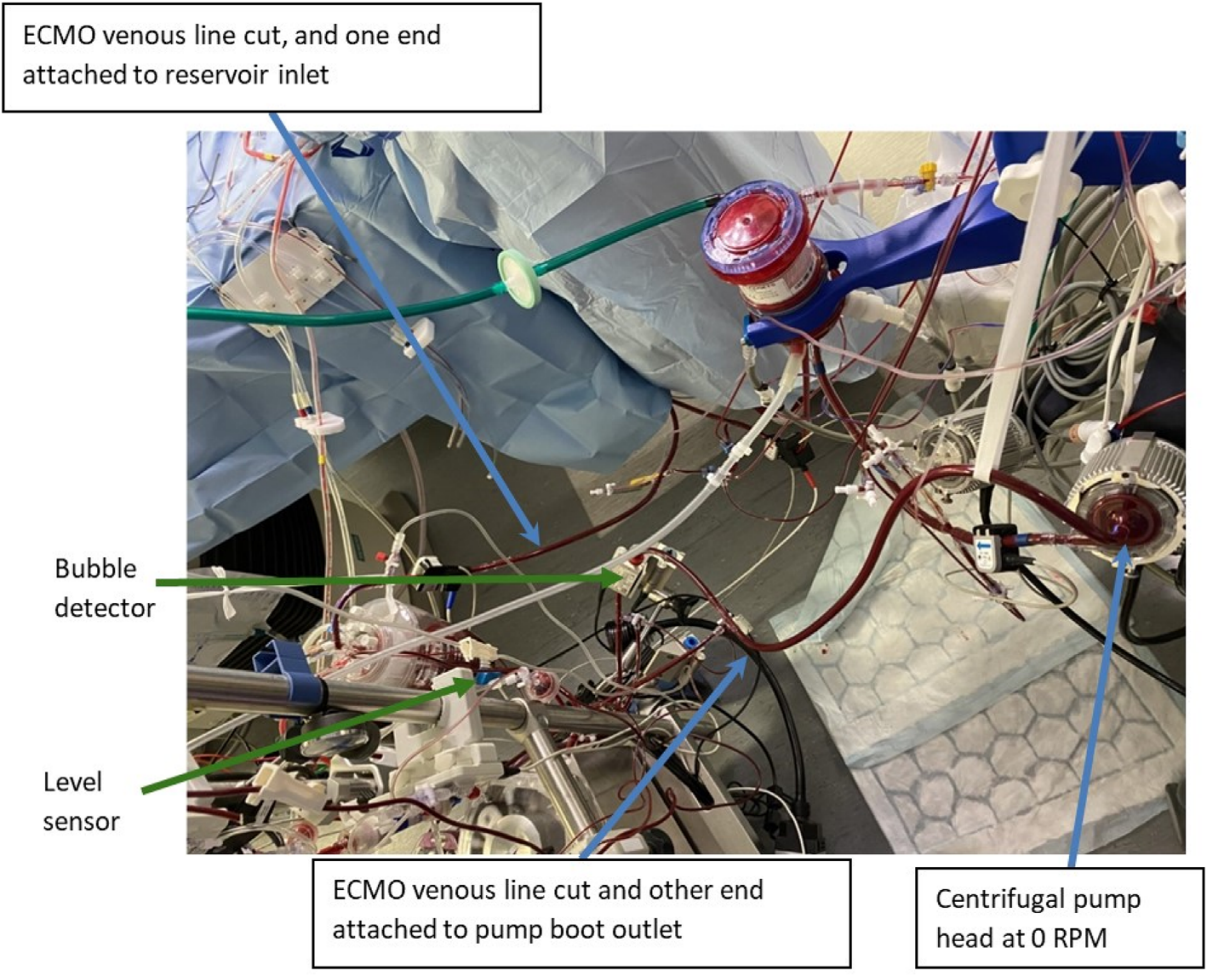
s5 MES circuit technique.

Again, the patient was fully heparinized prior to CPB conversion, with the same cannulation as patient one. The process was similar to the Quantum MES conversion; however, the lower limb of the venous line was attached to the roller pump outlet (Figs. 4 and 5). As of note, the CentriMag console power remained on for the pump head to be magnetically levitated for optimum blood flow pathway.

In both cases, the patients were successfully separated from CPB and recovered in pediatric intensive care.

## Comment

Systemic inflammatory response syndrome (SIRS) from CPB has been widely documented, with possible causes being ischemia-reperfusion injury, operative trauma, and blood contact with artificial surfaces [6]. The benefit of modifying the in situ ECMO circuit for cardiac surgery, rather than converting to a new CPB circuit, reduces the total surface area and priming volume, thereby minimizing patient hemodilution (Table 1). Rat models have shown that higher priming volumes lead to significantly elevated levels of pro-inflammatory cytokines TNF-α and IL-6 [7]. Also, by reducing the foreign surface area, the expression of the leukocyte-specific receptor CD11b, as a marker of inflammatory response, is reduced [3]. Thus, utilization of the MES may attenuate the inflammatory response and its consequences. Using this strategy, pediatric intensivists commented on less post-operative bleeding and blood product requirements, potentially due to the reduced surface area and priming volume. Limiting blood product usage to a single unit of packed red blood cells (RBC), reduces the risk of immunological complications such as transfusion-related acute lung injury (TRALI) and febrile non-hemolytic reactions, as the prevalence of transfusion reaction in children is twice that of adults [8, 4, 9, 10].

**Table 1:** Preoperative priming volumes and patients’ demographics for both cases.

Patient No.	Weight (kg)	Estimated blood volume (mL)	Hematocrit prior to CPB (%)	Full CPB circuit volume (mL)	Estimated Hematocrit on CPB with no RBC’s added (%)	s5 MES (mL)	Estimated Hematocrit s5 MES with no RBC’s added	Quantum MES (mL)	Hematocrit on Quantum MES with no RBC’s added (%)
1	3.3	281	35	300	17	80	27	120	25
2	3.4	289	31	300	15	80	24	120	22

A comparison of the Quantum MES and s5 MES identified the following features below.

The Quantum MES used the CentriMag pump to control patient flow, with the safety feature of an arterial occluder linked to the bubble detector. The Quantum MES also allows for a smaller footprint in the theatre environment. Nonetheless, the limitations of this setup are: include perfusion control across both the ECMO cart and Quantum MES, no line pressure regulation, and no level sensor function. In the unlikely event of the reservoir emptying, the bubble detector located on the reservoir outlet and arterial line occluder would activate to prevent patient air. Although the circuit was without pressure regulation, an audible alarm would be activated within target pressure limits.

Using the s5 MES to carry out this procedure, the stated limitations of the Quantum MES were overcome. The s5 MES allowed the roller pump of the s5 HLM to control the blood flow and have a functional level sensor. By using the roller to control flow, the centrifugal pump head remained in situ; however, at a reduced pump speed, so it remained patent for potential conversion back to ECMO. The team needed to be aware that pressure regulation was monitored pre-oxygenator, rather than post-oxygenator, as in our conventional CPB circuit. Thus, circuit line pressure would measure considerably higher.

Further modification of the s5 MES could be carried out by dividing the venous line between the pump head and the oxygenator of the ECMO circuit. This would enable kinetic-assisted venous drainage via the centrifugal pump head. However, this was not possible with our current ECMO circuit design, due to multiple connectors in this position.

This report shows that converting the patient from ECMO to CPB is not without its risks; however, by using this technique the extensive foreign surface exposure is reduced, along with the use of blood products and their associated detrimental effects. This novel strategy also has the flexibility of conversion back to ECMO after cardiac surgical revision, if required.

## Data Availability

All available data are incorporated into the article.
